# The effect of multispectral image fusion enhancement on human efficiency

**DOI:** 10.1186/s41235-016-0045-0

**Published:** 2017-03-20

**Authors:** Jennifer L. Bittner, M. Trent Schill, Fairul Mohd-Zaid, Leslie M. Blaha

**Affiliations:** 1 Air Force Research Laboratory, 711 HPW/RHCV, 2255 H Street, Wright-Patterson AFB, Dayton, 45433-7022 OH USA; 2 Ball Aerospace & Technologies Corp., 2875 Presidential DriveFairborn, 45324 OH USA

**Keywords:** Ideal observer analysis, Efficiency, Image fusion, Multispectral imagery, Landolt C

## Abstract

The visual system can be highly influenced by changes to visual presentation. Thus, numerous techniques have been developed to augment imagery in an attempt to improve human perception. The current paper examines the potential impact of one such enhancement, multispectral image fusion, where imagery captured in varying spectral bands (e.g., visible, thermal, night vision) is algorithmically combined to produce an output to strengthen visual perception. We employ ideal observer analysis over a series of experimental conditions to (1) establish a framework for testing the impact of image fusion over the varying aspects surrounding its implementation (e.g., stimulus content, task) and (2) examine the effectiveness of fusion on human information processing efficiency in a basic application. We used a set of rotated Landolt C images captured with a number of individual sensor cameras and combined across seven traditional fusion algorithms (e.g., Laplacian pyramid, principal component analysis, averaging) in a 1-of-8 orientation task. We found that, contrary to the idea of fused imagery always producing a greater impact on perception, single-band imagery can be just as influential. Additionally, efficiency data were shown to fluctuate based on sensor combination instead of fusion algorithm, suggesting the need for examining multiple factors to determine the success of image fusion. Our use of ideal observer analysis, a popular technique from the vision sciences, provides not only a standard for testing fusion in direct relation to the visual system but also allows for comparable examination of fusion across its associated problem space of application.

## Significance

The natural visual world is complex, varying in presentation over innumerable factors such as sunlight, shading, reflectance, and composition. Given this complexity, our human visual system is not always equipped to efficiently interpret all aspects of our surroundings. For example, identifying features of a scene at night may be extremely difficult. But, during the day, one may see so many features of the same scene that simple tasks become hard to accomplish. To combat such limitations in real-world applications, multispectral imagery is utilized to enhance particular aspects of the environment such as with near-infrared (i.e., night vision) and long-wave infrared (i.e., thermal, heat-intensifying) spectral bands. These types of visual enhancements are particularly important in applications of critical decision making, such as in military and law enforcement fields. Determining the most appropriate and effective imagery in aiding human vision, however, can be tricky, especially given that an image taken in one individual spectral band can distort important information otherwise captured in another spectral band. To take advantage of multiple vision enhancements, researchers have employed image fusion, a systematic combining of multispectral imagery. With such a variety of image enhancements all aiming to improve human vision, it is important to understand if and how the human visual system takes advantage of multispectral and fusion techniques. This requires testing of the impacts on the visual system at its most basic level to understand how efficiently information is processed over changes to image presentation. Our current paper addresses these critical research questions.

## Background

Researchers have long searched for ways to enhance human visual perception and performance. Given that the human visual system can be affected by varying characteristics of visual presentation, an area of critical interest in this field is the study of multispectral image fusion. Image fusion is a technique that takes two visual inputs (e.g., images captured in two different spectral bands) and algorithmically combines them in an effort to produce a vision-enhancing output image. The stated goals of fusion traditionally surround some improvement of human perception and/or computer processing, aiming to produce stimuli that are more informative and more suitable to visual perception, maximize relevant information particular to a task, increase perceptibility, and provide other such advancing effects (e.g., McCarley & Krebs, 2006; Toet et al., 2010). However, fusion’s inherent processes and its ultimate implementations encompass a large problem space of parameters of consideration to determine if these goals are being met.

There are many ways to fuse imagery, many types of imagery that can be fused, and many applications for its ultimate use. This means that testing the effectiveness of fusion not only requires comparison of its effects in relation to those of the *unfused* or component single-band imagery, but also requires an understanding of the impacts of the stimuli being fused, the fusion techniques implemented, and the relevant task or application for the fused imagery. Additionally, when fusion is intended for human use, as it is in many of its applications, the measurement of effectiveness must meet the standard of direct assessment of the human visual system in order to test the goal of enhancing human perception.

The current state of evaluation for the visual impact of image fusion lies primarily in the realm of image quality metrics (e.g., Hossny, Nahavandi, Creighton, Bhatti, & Hassan, 2013; Kekre, Mishra, & Saboo, 2013; Raut, Paikrao, & Chaudhari, 2013; Wang, Yu, & Shen, 2009) and user preference (e.g., Aguilar et al., 1999; Ryan & Tinkler, 1995), with only limited studies of experimental human performance with image fusion. This paper provides a more discerning examination of image fusion, assessing its direct impact on the human visual system by applying a technique commonly used in visual perception research: ideal observer analysis. Using this approach, we establish a foundation for studying the vast problem space that encompasses image fusion research and examine the impact of fusion and its component inputs on human information processing efficiency for a simple stimulus set and task. This directly addresses the main image fusion goals and allows for a better understanding of how enhanced imagery is affecting our visual system.

### Current image fusion testing and evaluation

**Fig. 1:**
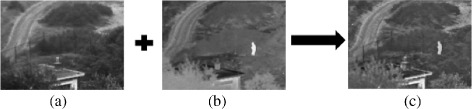
Example scene imagery captured in the **a** visible spectrum and **b** thermal (long-wave infrared *(LWIR)*) spectrum and **c** created through an image fusion algorithm. Individual sensor imagery is publicly available from the TNO Image Fusion Dataset (Toet, 2014). Imagery in this figure adapted from Toet, et al., (2010)

To initiate an understanding of the phenomenological impact of image fusion on vision, consider the example shown in Fig. 1. Figure 1 a shows a scene captured in the traditional visible spectrum. In this image, an observer can plainly see landscape details such as fences, trees, roads, etc. Capturing this same image in the long-wave infrared (i.e., thermal) spectrum provides a different set of salient features (Fig. 1 b). Here, a glowing human body, a component that may not have been detected in the visible image, is quickly recognized in the field. Note now, however, that this thermal image has lost much of the landscape details immediately apparent in the visible image. To reconcile these two sets, a fusion algorithm can be used to produce an image that shows both the landscape details as well as the glowing human (Fig. 1 c).

Applications of image fusion are intended to encompass a “best of both worlds” visual presentation. The enhanced imagery from fusion is generally assumed to be as good as or better than the corresponding counterpart images (Essock, Sinai, DeFord, Hansen, & Srinivasan, 2004); however, it cannot be ensured that fused images will always adhere to this standard. Image quality metrics constitute the most utilized fusion evaluation techniques (for reviews, see Hossny et al., 2013; Kekre et al., 2013; Raut et al., 2013; Wang et al., 2009). These metrics range over a variety of mathematical image processing principles but generally involve the measurement of some property of the fused image as it relates to how it was combined from the single-band image components (Hossny et al., 2013; Kekre et al., 2013; Raut et al., 2013; Wang et al., 2009). Common examples include mutual information, fusion symmetry, signal-to-noise ratio, entropy, root mean square error, and other such similar techniques (Hossny et al., 2013; Kekre et al., 2013; Raut et al., 2013; Wang et al., 2009).

Although these metrics may be of some value to the enhancement of computer vision or image processing, an immediate problem in regard to human visual perception is their lack of a direct relationship to the human observer. Most quality metrics measure variation in physical image properties only (e.g., pixel deviation, image intensity, contrast), without consideration for the impact of those properties on perception and/or decision. A small number of metrics have incorporated quality assessments that consider human visual system properties, such as the contrast sensitivity function (e.g., Chen & Varshney, 2007; Chen & Blum, 2009). Although a significant step in the connection between image fusion and human perception, even these types of image fusion evaluations disregard the potential impact of key elements such as task and stimulus content.

Consider again the example in Fig. 1, but now assume a task where an observer must detect a human target. Although our fused image (Fig. 1 c) provides an encompassing combination of the salient features from the individual sensors, the thermal image (Fig. 1 b) primarily highlights the human target without distracting scene features. Thus, for this task, it is possible that the most appropriate image enhancement may not be fusion at all, but the thermal component imagery instead. This vital consideration, that single-band stimuli may be more appropriate than the fused stimuli for given experimental parameters, is missed with nearly all quality metric applications. Moreover, many traditional metrics do not even allow for evaluation of the individual sensor. That is, many of them incorporate calculations based only on how the information was combined. Thus, the resulting measure can only be applied at the fused level without a test for the relative individual component sensor quality or performance.

To truly understand whether image fusion has an enhancing effect, it is necessary for the evaluation approach to consider the psychological factors (e.g., task, stimulus content, etc.) that can impact human visual performance. Thus far, efforts for assessment of humans lie in two areas: subjective rating studies and a small, very disparate set of research studies measuring basic human behavior. The former asks participants for rankings of characteristics ranging from pure preference of imagery up to self-ratings of their personal aptitude in workload, confidence, and ability while completing tasks using fused and unfused imagery (e.g., Aguilar et al., 1999; Ryan & Tinkler, 1995). These types of evaluations, although informative on the partiality of users, cannot ensure a verifiable measure of the impacts of imagery on perception, as human judgement of self-preference and performance provides many opportunities for internal error and/or bias. The latter studies do provide an understanding of human behavior with imagery in given contexts; however, this behavior is hard, if not impossible, to compare impartially across applications and techniques in order to address the overarching image fusion goals. More specifically, these studies, sparse in number, encompass a diverse scope of methods, analyses, and applications with measurement techniques that cannot compare the impacts on the human visual system across such variety without confounds from the variety itself (i.e., information content, see more on this in the ideal observer approach to fusion evaluation section below).

Tables 1 and 2 summarize the procedures and analyses used in the human behavioral research. The listed items vary in both complexity and structure within and between studies. The variety seen here is an initial indicator of the diversity in image fusion approaches. All of this research has some goal of examining the impact of fusion; however, aspects such as stimulus content, fusion type, and experimental focus vary from study to study. For brevity, we will not summarize all human performance studies, but will give a few examples elucidating the range of applications.

**Table 1:** Procedures utilized in research examining human performance with image fusion

Procedure	References
Target detection/	Krebs, Scribner, Miller, Ogawa, and
localization	Schuler (1998); Krebs and Sinai (2002);
	McCarley and Krebs (2000); Neriani et al. (2008);
	Steele and Perconti (1997); Waxman et al. (1996)
Identification/	Essock et al. (1999); Essock et al. (2004);
categorization	McCarley and Krebs (2006); Sinai, McCarley,
	Krebs, and Essock (1999); Steele and
	Perconti (1997); Toet and Franken (2003)
Discrimination	Krebs and Sinai (2002)
Relational positioning	Toet et al. (1997)
Inversion	Krebs and Sinai (2002); Sinai et al. (1999);
	Toet and Franken (2003)
Horizon assessment	Steele and Perconti (1997); Toet and Franken (2003)
Passive viewing	Toet, de Jong, Hogervorst, and Hooge (2014)

**Table 2:** Analyses utilized in research examining human performance with image fusion

Analyses	References
Reaction time/	Essock et al. (1999); Essock et al. (2004);
accuracy	Krebs et al. (1998); Krebs and Sinai (2002);
	Neriani et al. (2008); Sinai et al. (1999);
	Steele and Perconti (1997); Toet et al. (1997);
	Waxman et al. (1996)
Signal detection	Krebs and Sinai (2002); McCarley and Krebs (2000);
measures	McCarley and Krebs (2006); Sinai, DeFord,
	Purkiss, and Essock (2000); Toet and Franken (2003)
Free recall measures	Toet et al. (2014)
Eye tracking fixations	Toet et al. (2014)
Feature tracing	Toet et al. (2010)

In Neriani, Pinkus, and Dommett (2008) participants were asked to view terrain boards captured in visible and thermal spectra and fused via four fusion algorithms. The task consisted of deciding whether a “hot” tank was present or absent and then identifying in which quadrant it appeared. Reaction time results showed no significant improvement while viewing fused imagery. Krebs and Sinai (2002) also used a basic visual task structure, but examined chromatic and achromatic fused imagery of real-world nighttime scenes containing woods, fields, roads, and buildings. Over three experimental investigations, one where participants reported whether people or vehicles were present in the scene, one where the task was to determine if a scene was upright or inverted, and one where participants indicated whether two image presentations were the same or different, researchers found varying effects of fusion with strong task dependencies. Essock and colleagues (Essock, Sinai, McCarley, Krebs, & DeFord, 1999; Essock et al., 2004) took a categorization approach to their tasks, asking participants to classify whether stimuli represented imagery types with content such as sky, trees, buildings, and water. In these studies, varying patterns in *d* ^′^ were found across categories for monochrome fusion and individual sensor types; however, color fusion produced better performance than its individual sensor counterparts overall.

Other studies have taken a more applied approach to fusion testing. Toet, IJspeert, Waxman, and Aguilar (1997) asked participants to evaluate the position of a person relative to scene detail in still images taken from various frames of video. Participants were instructed on specific scenarios for each video: (1) monitoring a fence while guarding a United Nations camp (determining position in relation to a fence in order to distinguish innocent bystanders from those wanting to perform subversive action), (2) guarding a temporary base in a wooded area (determining position in relation to the trees to detect and counter infiltration attempts), and (3) surveying a large dune landscape (determining position in relation to dunes to detect any attempt to infiltrate a certain area). In this study, both color and grayscale fusion produced less error in the response of a target’s relational position than the images from the single-band counterparts. Steele and Perconti (1997) selected night vision-qualified Marine Corps and Army National Guard rotary wing aviators for their participants in a part task simulation to examine the impact of image fusion. Tasks widely varied in this study, with questions specifically related to the stimulus content. These included locating objects, determining positions, giving details about objects, determining if the horizon was level, identifying shapes and orientations, voting on acceptability of images, and giving rank orders. Results were mixed, varying by type of fusion, task, and scene content. Finally, there is a small, scarce set of studies that examine image quality metrics in relation to direct human performance (Howell, Moore, Burks, & Halford, 2007; Wei, Kaplan, & Burks, 2009). For example, Howell et al. (2007) correlated human performance ratings in an object identification study with image fusion quality metrics findings. Ultimately, these researchers determined that in their set of examinations, no particular metric had the best correlation.

The full set of human performance studies provides an exploration into the impact of fusion when applied to specific contexts and experimental structures. Collectively, the research provides inconsistent results. The source of this variability most likely originates in the disparate scope of image fusion specifications, applications, analytic techniques, and procedural methods used across studies. This paper uses ideal observer analysis to establish a framework that allows for comparison across such factors while accounting for the inherent amount of information content in the full image fusion application. Additionally, we test this on a simple stimulus and task experimental structure to understand the basic impacts of fusion on the visual system.

### Ideal observer approach to fusion evaluation

Image fusion appearance can be considerably affected by the characteristics of its combination such as sensor components, combining algorithm, environmental collection conditions, and stimulus content (e.g., Krebs & Ahumada Jr., 2002). Additionally, the effect of fusion on the visual system has great potential to be influenced by changes in task constraints, stimulus attributes, and observer characteristics. Thus, understanding fusion’s overarching impact on the visual system can be tricky given the potential for information to change as the parameters of its implementation change. To provide a direct comparison of the impacts of different fusion enhancements on the visual system thus requires consideration of how information changes across imagery.

We employ ideal observer analysis (e.g., Geisler, 1989, 2011) to examine the effect of varying single-band and fused imagery enhancements on information processing in the human visual system. This powerful technique from the vision sciences (see Geisler, 2011 for review) examines human performance in relation to a statistically optimal Bayesian decider (i.e., an ideal observer). The ideal observer makes use of all information within a given experimental structure, allowing for it to establish a strict upper bound on performance and operate at 100% efficiency. The derivation of the ideal decision rule takes into account all factors of what is being examined (i.e., stimuli, task constraints, and any other experimental manipulation). Thus, the performance of the ideal observer is indicative of the relative amount of information across various experimental manipulations. In our experimental design, ideal observer simulations were performed over blocks of trials that varied in single-band and fused imagery enhancement. Human performance was examined in relation to the ideal observer, a construct defined as *efficiency*, to determine the direct impact of each of these imagery types.

The use of ideal observer analysis provides a number of distinct advantages to understanding the impact of multispectral and image fusion enhancements. Specifically, it allows us to directly measure human information processing without the construct of information content. This is vital to understanding the effects of imagery on the human visual system while taking into account the information variation over experimental factors such as stimulus and task complexity. With this, we are able to directly address the overarching goals of image fusion and image enhancement and examine the multidimensional problem space. Additionally, the ideal observer provides an in-depth look at the variation in information distributions over imagery enhancements. This can be beneficial to guiding future human experimentation. This important characteristic is covered in more detail in the Discussion.

## Experiment

We provide in this paper a study of the impacts of image fusion using ideal observer analysis. The goals are twofold: (1) to establish a framework for testing the impact of image fusion on human information processing efficiency over the varying aspects surrounding its implementation (e.g., stimulus content, task) and (2) to examine the effectiveness of fusion in a basic application. Within the experimental investigation we evaluate the core influence of single-band imagery and image fusion enhancements on the human visual system with a simple experimental structure. Specifically, we examined a simple 1-of-8 identification task on the orientation of Landolt C images presented over varying imagery conditions. Through the derivation of an ideal observer, we examined the relative amount of information between imagery sets. We then calculated human efficiencies through the relationship between human and ideal performance.

To consider fusion as a whole, testing must be approached systematically, building from fundamental examinations to more complex applications with direct comparison of results at each step and consistent consideration for the impacts on the human visual system. This requires that we start with basic, yet exemplary, experimentation in which the imagery captured fits within a well-defined, simple task structure. The stimuli for our experimental conditions were methodologically chosen for the purpose of providing a simple structure that encapsulates the characteristics of single-band image enhancement and algorithmic fusion combination. Considering again the example in Fig. 1, this imagery, although interesting in application and important to the demonstration of fusion with natural scenes, provides a number of initial confounds for principled examination of the foundational impacts of multispectral and fusion enhancement. Specifically, an investigation using such stimuli could likely provide results that are skewed to the complex characteristics of the scene itself or to complicated task demands that accompany interaction with such imagery.

Starting instead with a simple stimulus allowed us to see if the goals of image fusion (i.e., enhancement) would hold given basic image content, as fusion is assumed many times to be as good as or better than its corresponding single-band images (Essock et al., 2004). The results of this experiment stand on their own for our chosen simple experimental structure, and the overarching process provides the framework for testing at all levels of the fusion multidimensional problem space.

## Methods

A total of six conditions (c0–c5) were used in this experiment. Each condition corresponded to a particular pairing of single-band imagery. Within each condition there were nine experimental blocks, two blocks corresponding to the single-band imagery types (e.g., in c0: visible, hot-white (HW) thermal) and seven blocks for each of the algorithmically combined image fusion sets (e.g., average, Laplacian, principal component analysis (PCA), etc.). All work was carried out in accordance with the Code of Ethics of the World Medical Association (Declaration of Helsinki).

### Participants

The study included a total of 28 participants (15 male, 13 female), ranging in age from 18 to 48. All participants were recruited from Wright-Patterson Air Force Base, Wright State University, and the surrounding area. Participants had normal to corrected-to-normal vision and unencumbered use of both hands. Informed consent was obtained from all individual participants included in the study. Twenty-four participants (four per condition) completed only one study condition. Four participants (Participants 1–4) completed all conditions in the study in order (c0–c5) to determine if there were measurable learning effects. The participants who completed all conditions initially consented for condition c0 alone and were invited to participate in all future conditions based on their availability.

### Materials

The experiment was performed using a 2012 Mac Pro running Mac OSX 10.6 attached to a VIEWPixx/3D display monitor made by VPixx Technologies Inc., St Bruno, QC, Canada. The monitor was set to 1920x1080 resolution with a 120 Hz refresh rate and was calibrated prior to experimentation using a Minolta CS-100 photometer. Responses were made with a numberpad on a standard keyboard. Participants completed the experiment seated in a dark room at a standard table with their chin in a chinrest positioned 140 cm from the computer monitor. The monitor was the only source of light during the experiment.

### Stimulus creation

We photographed Landolt C images in varying individual sensor bands in a controlled environmental setting and fused them over a number of traditional fusion algorithms. The imagery sets represent the most basic elements of each type of enhancement, allowing us to examine their core influence on human perception.

The Landolt C stimulus was chosen as an experimental target because it demonstrates the basic elements of single-band imagery while providing comparison across image sets. The stimulus itself, although very simple in form, exhibits the attributes that correspond to each particular type of visible enhancement including elements such as “glow,” camera noise, edge “sharpness”/“blur”/“detail,” etc., all characteristics that have the potential to influence human perception. Given the capture of such features, the ultimate fusion of these images provided a representation of the prime effects of each algorithm with a combination of basic single-band image capture.

**Fig. 2:**
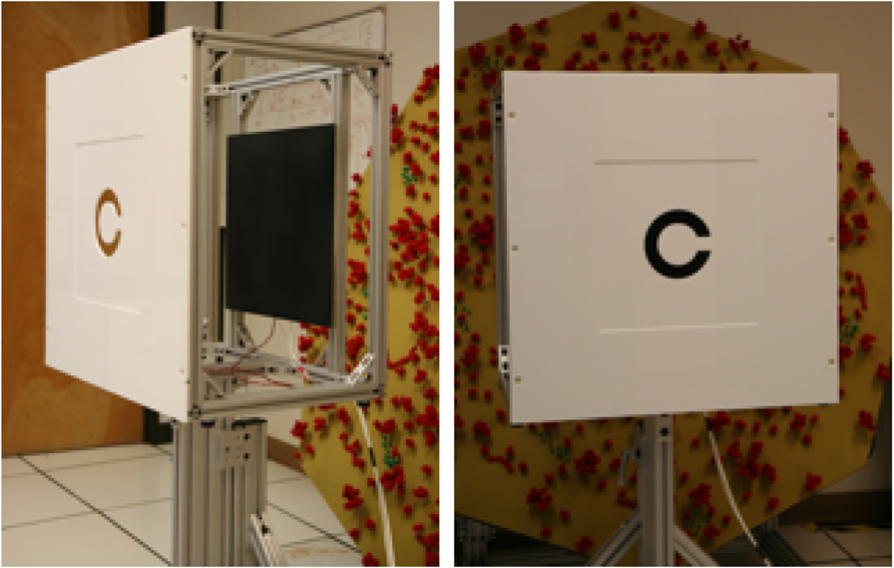
Landolt C apparatus. Images as in Pinkus, Dommett, and Task (2010)

**Capture**: Imagery for this study was obtained as part of a larger image collection utilizing a number of multispectral cameras. Table 3 provides the specifications of five of the cameras utilized in the large collection. The current study made use of the low resolution visible, night vision (NIR), short-wave infrared (SWIR), and hot-white (HW) thermal (LWIR) images. We also created hot-black (HB) imagery, digitally inverted from the HW images collected. This was included in our examination, as many LWIR cameras used in military and law enforcement fields include a physical switch option for HB or HW preference. The Landolt C photographed with each camera was constructed as a cut-out from a white acrylic sheet with a black heating plate as the background (heated for thermal image capture). Figure 2 shows this construction. Small metal squares (warmed by touch for thermal collection) were added to the outer corners of the Landolt C apparatus to provide reference for image registration. Images of the Landolt C were captured in eight orientations, 0°, 45°, 90°, 135°, 180°, 225°, 270°, and 315°, through physical rotation of the acrylic plate. Ten images were captured for each orientation within camera types.

**Table 3:** Camera specifications for image capture

Camera type	Spectral band	Array size	Collection
	(*μ* *m*)	(HxV)	conditions
Visible (low resolution)	0.4−0.75	640 x 480	Sunlamp *@*80W
Visible (high resolution)	0.4−0.75	768 x 494	Sunlamp *@*80W
Night vision (NIR)	0.665−0.9	768 x 493	Sunlamp *@*10W
SWIR	0.9−2.5	320 x 240	Sunlamp *@*40W
Hot-white	7−15	640 x 480	No lights;
thermal^a^ (LWIR)			heatplate *@* *Δ*10°C

^a^Digitally inverted to create hot-black thermal imagery

NIR, SWIR, LWIR stand for near, short-wave, and long-wave infrared, respectively; sunlamp settings in wattage

Because of the differing physical compositions and functions (e.g., night or day use, capture capabilities) of single-band sensor cameras, image capture condition variables such as lighting, zoom, and distance may never be held to strictly equivalent levels without adverse effects (e.g., extreme amounts of noise in the image, damage to the camera due to excessive light exposure, Landolt C appearing too small or out of frame). Therefore, our goal was to control as many environmental factors as possible to use the most equivalent set of capture conditions across the various sensors while maintaining a well-calibrated image in each spectral band. Thus, a concerted effort was made to provide as much consistency and control as possible across sensors with a discernible Landolt C within each camera. All images were collected in the same room using the same Landolt C apparatus. The distance between the camera and apparatus, camera zoom, and focus were set for each condition such that the Landolt C resided centrally and was of a similar size in relation to the image frame (see the following Registration section for more information on post-capture image sizing and cropping). A sunlamp suspended from the ceiling at a height of approximately 120 inches with adjustable wattage settings was used in all conditions requiring lighting. Wattage values were chosen such that the image appeared clearly and with minimal stimulus noise for each camera. These values are listed in Table 3.

**Registration**: Careful registration of pre-fused imagery is essential to producing fused images free of extraneous artifacts in any fusion application. Given that our imagery was collected with cameras of varying geometries and that stimulus orientations were produced through physical rotation of the Landolt C apparatus, the potential for misalignment of raw imagery existed from a number of error sources. Therefore, the registration process implemented in our study required assessment of alignment both *within* and *between* sensor stimuli.

**Fig. 3:**
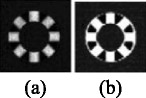
Example images resulting from the cumulative absolute squared difference between all stimuli within a sensor set. Sets resulting in difference images like those shown in **(a)** did not require within-sensor registration. Sets resulting in difference images such as those in **(b)** were required to go through the process of within-sensor registration

For selecting the sensor types that required within-sensor registration, the cumulative absolute squared difference between the 80 stimuli was calculated for each sensor set. With this technique, images of perfect alignment over orientations produced a difference image showing clear portions of all eight orientation “gaps” (i.e., the circular portion of the Landolt Cs cancelled out across stimuli). Difference images for each sensor set were calculated and examined visually for this property, and those deemed to have differences outside of the structure were further subjected to within-sensor registration. Figure 3 provides examples of this determination.

Alignment *within* sensor sets encompassed matching all Landolt C orientations from a particular sensor to the first “up” image taken in that set. This anchor image provided a basis for position for all Landolt Cs within that set. Using the similarity method, each image was matched to the within-sensor anchor through both translation and rotation, thus aligning all images on top of each other within that set.

Registration *between* sensor sets was performed on all imagery regardless of within-sensor registration determination. This process also required the use of an anchor image. To ensure that all imagery was aligned consistently across sensors, the anchor image for between-sensor registration was the first “up” image taken in the low resolution visible set. This provided a basis for defining not only the position of the Landolt C (as in within-sensor registration) but also the location, size, and proportion of the stimulus in relation to the image frame. Between-sensor registration used the projective method allowing for shifts of the imagery to match any difference in viewing angle, thus equating all stimulus locations regardless of camera geometry. This method was applied to all stimuli following any already-completed within-sensor registration.

During both methods of registration, matching of each image to the chosen anchor encompassed alignment of a set of registration points via the chosen similarity and/or projective method. Four of the registration points used were the registration markers placed on the outer square of the Landolt C apparatus during image capture. The other points were derived from the *imfindcircles* function in MATLAB, which uses the circular Hough transform to detect the circular portions of the Landolt Cs. Following within- and between-sensor registration, all images were cropped to 150x150 pixels.

**Fusion**: Image fusion was accomplished using the Image Fusion Toolbox for MATLAB 5.x version 1.0 (Rockinger, 1999) which encompassed a set of six traditional fusion algorithms: average, Laplacian pyramid (Laplacian), minimum, maximum, principal component analysis (PCA), and discrete wavelet transformation (DWT). A seventh function was added after we noticed a number of cases where PCA produced uninterpretable imagery (see, for example, the c4 PCA in Table 4). Further investigation of these phenomena revealed that the traditional PCA algorithm allowed for the resultant imagery to contain pixel values outside of the displayable range. Thus, the resulting images contained pixel values cut off at full white or full black values. Therefore, we created and additionally tested an adjusted PCA algorithm that rescaled the component scores to displayable values. See the Appendix for a further description of each fusion algorithm process.

**Table 4:**
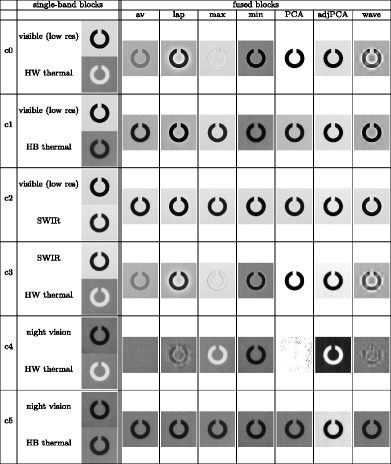
Sample imagery from each experimental condition (c0-c5) and block

Stimuli, previously registered and cropped, were fused such that matching orientation and image capture order were preserved during fusion. For example, in condition c0, the first image taken in the “up” orientation in the visible (low resolution) sensor was fused with the first image taken in the “up” orientation in the HW thermal sensor, and so forth. Sample images for each individual sensor and fused set are shown in Table 4.

**Final specifications**: Following all processes, stimulus sets of both fused and individual sensor images were adjusted to represent pixels in values of contrast relative to a background of average luminance using a contrast definition of (*L*_*pixel*_−*L*_*background*_)/(*L*_*background*_). Final stimuli were 4.1 cm x 4.1 cm, subtending 1.68° of visual angle horizontally and vertically. For the use of ideal observer analysis, stimuli were presented in white noise during experimental trials. This noise was uniquely sampled Gaussian contrast noise (*σ* ^2^=.01) added to each pixel of the image on each trial.

### Procedure

Table 4 depicts the condition and block types over the full experiment with an example from each imagery set. Participants 1–4 completed all experimental conditions in order (c0–c5) with block orders randomized within each condition. All other participants were randomly assigned to one condition, also with block orders randomly presented. Conditions were completed across two experimental sessions, with each session lasting approximately 90 minutes. Conditions c0–c3 and c5 contained nine blocks of trials each, while condition c4 contained only eight blocks, given that PCA did not produce discernible Landolt C imagery. Each of the blocks within conditions consisted of 350 trials.

At the beginning of a condition, participants were given a basic safety briefing and screened using the Snellen eye chart to ensure 20/20 corrected vision or better. A set of 24 practice trials was then administered to familiarize participants with the response selections. During these practice trials, a large outlined C-like shape was presented in one of eight possible orientations, with each orientation shown three times randomly across the practice trial set. The outlined object appeared on the screen for unlimited viewing until the participant responded. Feedback was then given in the form of a high (correct) or low (incorrect) beep.

Prior to experimental trials, participants were put through a 5-minute period of dark adaptation. Trials were self-initiated such that an outlined box was presented on screen until a button press by the observer. Following trial initiation, the stimulus, chosen randomly from the set of 80 possible images, was presented in Gaussian (white) noise for 500 ms. A 1-of-8 orientation response was made using the number pad on the keyboard, selecting the number key around the central ‘5’ which corresponded to the eight orientations of the Landolt C opening. Following response, auditory feedback (i.e., high, low beep) informed the participant of a correct or incorrect response.

### Threshold measurement

Performance in each block was defined through determination of a contrast energy threshold. Contrast energies (integrated squared contrast, measured in degrees squared) were varied over two interleaved staircases, a 1-down, 1-up and a 2-down, 1-up rule, for a total of 350 trials per block. A Weibull psychometric function was fit to the collective staircase data to produce a 50% contrast energy threshold (Note: the chance performance for a 1-of-8 identification task is ∼ 13*%*). Variability for each threshold was determined through 200 bootstrap simulations (Efron & Tibshirani, 1993).

### Ideal observer

Like human experimentation, the ideal observer performance for each block was simulated over a 1-up, 1-down staircase procedure to obtain a contrast energy threshold. The decision made by the ideal observer was formulated using Bayes’ rule in the given Landolt C task in a manner consistent with traditional ideal observer analysis application. This was accomplished by the following procedure.

On any trial, let *O* represent the orientation of the Landolt C and *S* be the noisy stimulus shown on a trial. In this task, the observer must decide between eight possible orientations (*i*=1,…,8) and each orientation has a set of ten images (*j*=1,…,10) that can be selected as the stimulus. The posterior probability for each orientation, *O*_*i*_, becomes:

$$\begin{array}{@{}rcl@{}} P(O_{i}|S) = \frac{P(O_{i})P(S|O_{i})}{P(S)} \end{array} $$

Given our experimental parameters, the prior probabilities for each orientation, *P*(*O*_*i*_), and the normalizing factor, *P*(*S*), are both constants that can be removed without affecting the relative orderings of *P*(*O*_*i*_|*S*). The probability of concern then becomes *P*(*S*|*O*_*i*_). Given that the stimulus is presented in Gaussian noise and there are ten possible images for each orientation,

$$\begin{array}{@{}rcl@{}} P(S|O_{i}) = \sum_{j=1}^{10}\prod_{k=1}^{n}\frac{1}{\sqrt{2\pi\sigma^{2}}}e^{-\frac{1}{2\sigma^{2}}(S_{k}-O_{ijk})^{2}} \end{array} $$

where *n* is the total number of pixels and *σ* is the standard deviation of the Gaussian distribution from which the external noise was generated. The ideal observer then chooses the *O*_*i*_ with the highest probability.

Ideal observer simulations for each block were completed over 10,000 trials. Just as in the human analyses, a 50% contrast energy threshold was found by fitting a Weibull psychometric function to the ideal staircase data and determining variability over 200 bootstrap simulations (Efron & Tibshirani, 1993).

### Efficiency

Efficiencies were defined as the ratio of ideal to human contrast energy threshold. A separate efficiency was computed for each single-band and fused image block within each condition. All estimates were computed at the individual participant level.

## Results

**Fig. 4:**
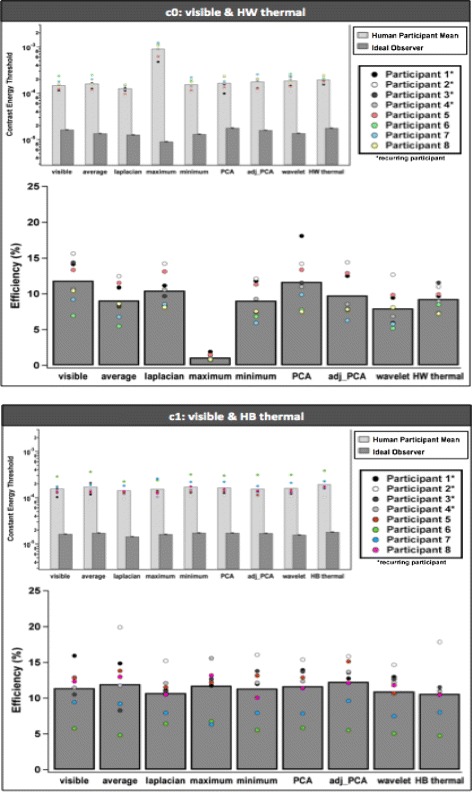
Results c0 and c1: Each box represents one experimental condition. Within each box are contrast energy thresholds graphs *(upper left)* and human efficiency *(bottom large)*. Bars of human data are means across individual participants, represented as *points*. Error bars on the ideal data are ± 1 *SD* derived from bootstrap simulations

**Fig. 5:**
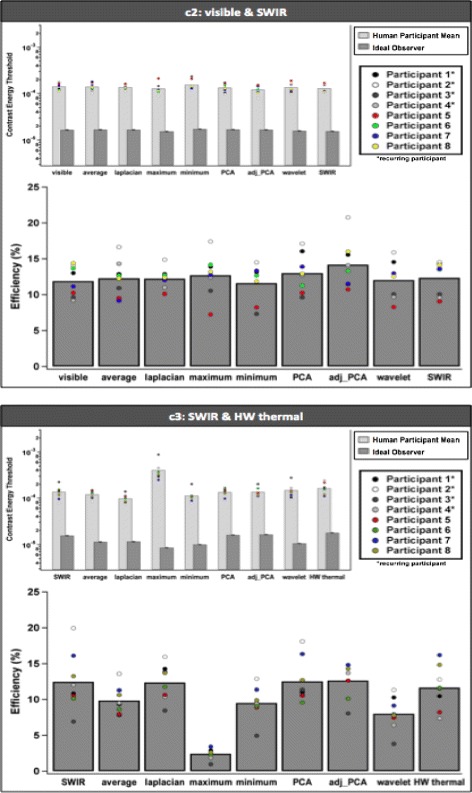
Results c2 and c3: Specifications are as noted in Fig. 4

**Fig. 6:**
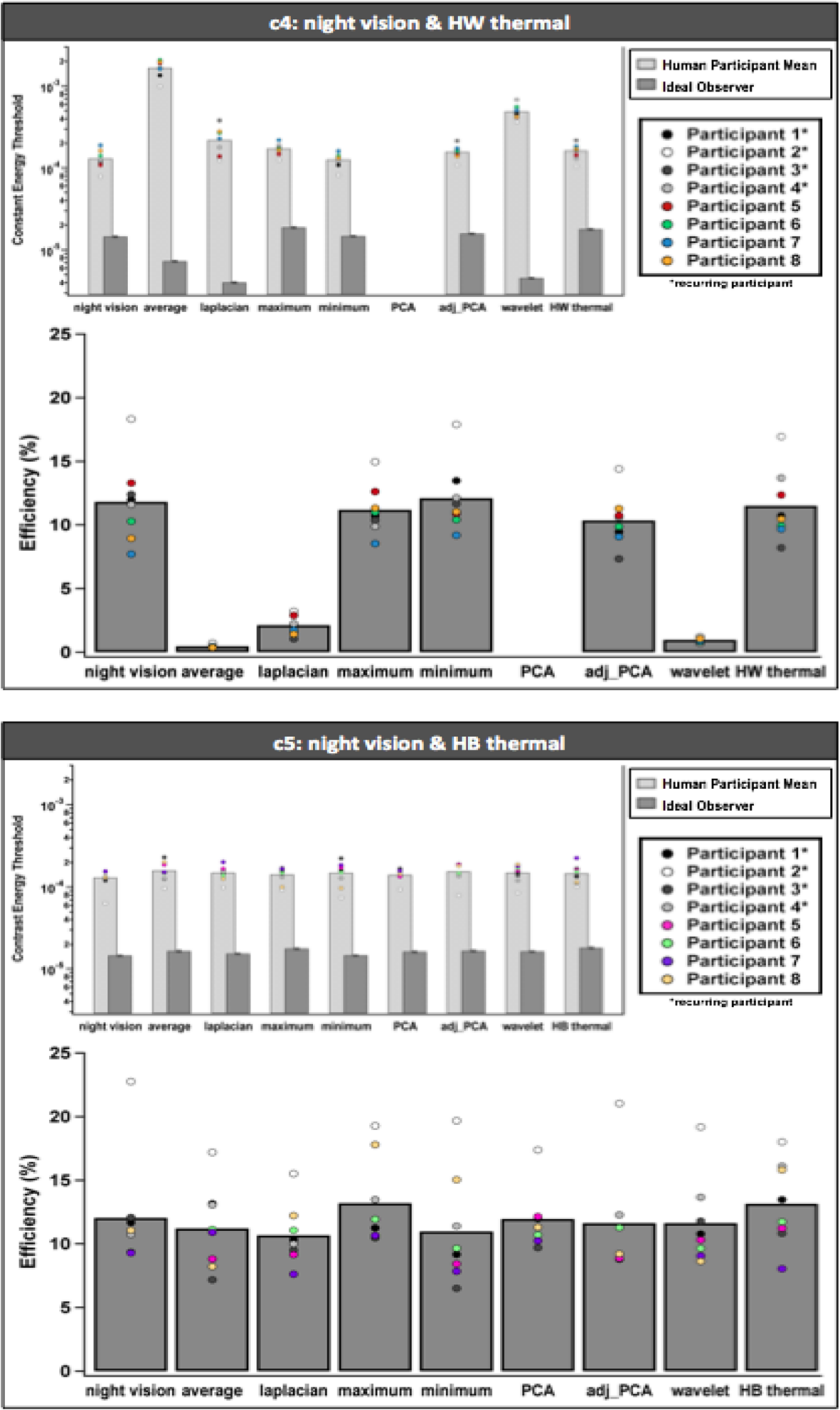
Results c4 and c5: Specifications are as noted in Fig. 4. Note: In condition c4 graphs, a single participant outlier was removed in both the average and wavelet conditions for proper viewing

Figures 4, 5, and 6 show the human and ideal performance over the six experimental conditions (i.e., visible-HW thermal, visible-HB thermal, visible-SWIR, SWIR-HW thermal, night vision-HW thermal, and night vision-HB thermal). Displayed within each condition box are two series of bar plots—one showing human threshold data and the results of the ideal observer simulations, and one showing human efficiencies. All single-band imagery data are represented in the outermost bars of each bar plot with the seven innermost bars representing the performance on the fused images derived from the two sensors on the ends.

### Contrast energy thresholds

The upper left graphs in each condition box contain contrast energy thresholds obtained from human experimentation and ideal observer simulation for each experimental block. Human thresholds indicate raw performance with each imagery type. Ideal observer thresholds are indicative of the relative amount of information between the block types with lower thresholds meaning more information.

We statistically examined the patterns in human contrast energy thresholds by applying a linear mixed effects model with participant as a random effect, over the dataset from the full experiment. Linear mixed effects modeling is useful for analysis of experiments with repeated measures, as is the case for our experiment. The analysis showed significant main effects of condition type (*F*(5,378)=12.35,*p*<0.0001,*η* ^2^=.04) and block type (*F*(11,378)=5.90,*p*<0.0001,*η* ^2^=.13), indicating that choice of single-band sensor combinations and imagery type were influential to the results. To examine the driving forces behind these differences, we investigated the relationship between imagery blocks within each experimental condition. A one-way repeated-measures analysis of variance (ANOVA) showed that there were significant differences between blocks in each condition except c5, with c0 (*F*(8,56)=59.06,*p*<0.0001,*η* ^2^=.84), c1 (*F*(8,56)=3.14,*p*=.005,*η* ^2^=.05), c2 (*F*(8,56)=2.40,*p*=.026,*η* ^2^=.10), c3 (*F*(8,56)=16.79,*p*<0.0001,*η* ^2^=.60), c4 (*F*(7,49)=4.71,*p*=0.0004,*η* ^2^=.35), and c5 (*F*(8,56)=1.13,*p*>.25,*η* ^2^=.05).

Post hoc pairwise comparisons using Bonferroni correction were performed between all block combinations within each condition set to determine which specific blocks differed from one another. These revealed significant differences between the maximum algorithm and all other blocks (respectively), and the Laplacian and hot-white thermal blocks within c0, and between the night vision and maximum, and maximum and minimum blocks within c4. No other differences between block types were shown to be significantly different within conditions in pairwise comparisons, meaning that the significant difference indications in the ANOVA in conditions c1, c2, and c3 indicate negligible effects, if any.

The dark gray bars presented in the contrast energy graphs of Figs. 4, 5, and 6 show the thresholds obtained in ideal observer simulations. Note that the amount of information provided to the human observer was not the same within and between all imagery conditions (i.e., ideal observer thresholds vary over blocks). Therefore, conclusions of the effects of image enhancement based on the human contrast energy threshold data should be made with extreme caution. Although the thresholds are representative of raw human performance, it is impossible to determine from these data alone whether the effects are driven by human ability to process information efficiently in the visual system or by differences in information content across imagery conditions. We must instead consider these data in relation to the inherent information content to determine human information usage across imagery types.

### Efficiencies

To directly measure human ability to use available information in each experimental block, we calculated human processing efficiencies. These were determined as the ratio of ideal to human contrast energy thresholds. These are displayed in the efficiency graphs in Figs. 4, 5, and 6.

As with the thresholds, we ran a linear mixed effects model with participant as a random effect over the full experimental dataset to determine if the condition type and block type were statistically significant across all participants. We again found significant main effects of condition type (*F*(5,378)=18.73,*p*<0.0001,*η* ^2^=.11) and block type (*F*(11,378)=7.46,*p*<0.0001,*η* ^2^=.10). We examined these effects further using a one-way repeated-measures analysis of variance with post hoc pairwise comparisons using Bonferroni correction between all block combinations within conditions. These crucial results are summarized in Table 5.

**Table 5:** Results from repeated-measures ANOVA and pairwise comparisons with Bonferroni correction on human efficiencies

Condition	Repeated-measures	Pairwise comparisons
	ANOVA	with significant differences
c0	F(8,56) = 40.75	every c0 block - maximum
Fig. 4 top graph	p < 0.0001	(respectively)
	*η* ^2^ =.62	visible-average
	significant	visible-wavelet
		Laplacian-wavelet
c1	F(8,56) = 1.24	N/A
Fig. 4 bottom graph	p = 0.296	
	*η* ^2^ =.03	
	not significant	
c2	F(8,56) = 2.33	no significant differences found
Fig. 5 top graph	p = 0.031	(i.e., differences, if exist, are
	*η* ^2^ =.08	negligible)
	significant	
c3	F(8,56) = 31.28	every c3 block - maximum
Fig. 5 bottom graph	p < 0.0001	(respectively)
	*η* ^2^ =.62	Laplacian-average
	significant	Laplacian-minimum
		Laplacian-wavelet
		wavelet-adj _PCA
c4	F(7,49) = 110.33	every c4 block - average
Fig. 6 top graph	p < 0.0001	(respectively)
	*η* ^2^ =.87	every c4 block - Laplacian
	significant	(respectively)
		every c4 block - wavelet
		(respectively)
c5	F(8,56) = 1.91	N/A
Fig. 6 bottom graph	p = 0.077	
	*η* ^2^ =.06	
	not significant	

Here, it was shown that conditions c1, c2, and c5 had no significant differences among image types (i.e., blocks). However, conditions c0, c3, and c4 showed that differences existed within the conditions. This is summarized in the middle panel of Table 5. Pairwise comparisons determined which specific blocks exhibited these differences. These are shown in the rightmost column of Table 5. Notably, conditions c0 and c3 shared the result that the maximum algorithm differed from all other image blocks within those sets. Additionally, c4 showed differences between all blocks with the average algorithm block, Laplacian algorithm block, and wavelet algorithm block.

Taken together, these results show that efficiencies are mainly affected by condition. That is, the choice of single-band sensor combination influences the variation in efficiencies across image type. Additionally, although some algorithms produced significant differences within conditions, no patterns were shown consistently across conditions due to algorithm choice, and fusion as a whole was not shown to outperform individual single-band imagery. The implications of these results will be considered further in the Discussion.

### Potential learning effects

Given the basic nature of the stimuli and task structure, the potential for an influence of learning existed over the course of the experiment. To examine if this was a factor in our data, an analysis was performed on the efficiencies from participants who completed all conditions in sequential order (i.e., Participants 1–4). Recall that each of these participants completed the full set of experimental conditions, c0–c5, with blocks assigned randomly within conditions. Given that these participants completed a total of 53 blocks, their extensive experience with the experiment allowed us to examine the potential for efficiency to be influenced over time in the study.

To examine these data, we arranged each participant’s efficiency by the order of blocks they completed over the course of the experiment. We then performed a linear regression on each participant’s efficiency values against their block order, calculating a regression slope. This analysis revealed that each participant’s regression slope did not significantly differ from zero, using an alpha of 0.05, meaning there was no significant increase or decrease in efficiency over block order. This suggests that, although we were using a simple experimental structure, there was no strong evidence of learning over time in the study.

## Discussion

In this paper, we used ideal observer analysis to examine the fundamental impact of single-band imagery and image fusion on the human visual system. This investigation took an approach that allowed for direct evaluation of human vision and gave a straightforward comparison of the varying features of fusion to address and evaluate the goals of image enhancement. The application of ideal observer analysis to image fusion provided an assessment that accounted for the information inherent in the stimulus and task at hand. By deriving efficiencies as a relation of human and ideal performance, we were able to objectively compare human information usage across image types without the confounding variable of information content. Additionally, the progression of experimental conditions over a simple experimental structure provided foundational-level analysis of the impacts of fusion and its associated properties while forming a framework for future investigations.

We found interesting patterns within our experimental exploration with a simple stimulus, task, and condition structure. Namely, (1) contrary to image fusion goals, fusion was not shown to be more effective as compared to its single-band counterparts on human efficiency; (2) there was no strong pattern of specific algorithm impact across conditions, meaning the choice of algorithm alone did not determine success or failure of fusion; instead, (3) the chosen spectral band combination appeared to drive varying result patterns. These findings are important to the underlying goals of fusion and future research. We will explore each of these as follows.

Our first major result showed that image fusion did not consistently provide an improvement to human visual processing over the single-band source images. In fact, we found that images taken from the single-band sensor cameras produced equivalent or, at times, better efficiencies than those from the various fusion combinations. This is vitally important, given that fused imagery is generally assumed to be as good as or better than its corresponding single-band imagery (Essock et al., 2004). Our finding highlights the importance of considering the individual sensor sets as part of the fusion evaluation and invalidates the assumption that all image fusion is enhancing. Where traditional image fusion evaluations fail to address the impacts of single-band imagery, either in inability to calculate this comparison (i.e., as in many traditional image quality metrics, see Hossny et al., 2013; Kekre et al., 2013; Raut et al., 2013; Wang et al., 2009) or through lack of consideration for the impact of the individual fusion counterparts, ideal observer analysis provides the flexibility to incorporate this comparison while directly examining human vision. Given that fusion aims to produce a more informative image, our experiment shows that neither this nor efficient usage of information with fusion can be explicitly assumed with its application.

Our study also examined seven basic fusion algorithms. Image fusion researchers often focus directly on finding the overall best algorithm of fusion. Looking at each of our algorithms individually, we found that no single algorithm choice produced a consistent benefit to efficiency over single-band imagery across our study. In fact, many algorithms varied in their impact across conditions, at times producing very poor efficiency values. Algorithm development is an important aspect of the study of image fusion. Our results show the need for considering additional factors beyond just that of algorithm choice when considering the perception goals of fusion. Our basic experimental structure shows that a specific algorithm cannot always be relied on to impact visual performance in the same manner over changes to other fusion factors such as stimulus content, task, or single-band sensor combination.

Note that, although we picked a set of traditional techniques, there are many other ways that researchers have fused imagery beyond the seven algorithms considered here (see Krishnamoorthy & Soman, 2010, for review). These range from expansions of the basic algorithmic equations (e.g., Krishnamoorthy & Soman, 2010) to colorization and/or color fusion (e.g., Toet & Hogervorst, 2012). Additionally, researchers have considered manipulation of basic physical properties, such as contrast, prior to and after fusion (e.g., McCarley & Krebs, 2006). These types of extensive combination techniques were beyond the scope of our investigation and were thus not considered here; however, it is important to note that our framework can be adjusted to examine such manipulations in relation to the human visual system. Given that ideal observer performance is stimulus dependent, the expansion of our setup to include other image manipulations is as simple as including those manipulations in the computational “templates” of the ideal observer and experimental study of human data. In this way, various other image properties can be examined and compared in the future with respect to human efficiency.

Finally, the biggest impact on efficiency in our investigation was in relation to changes to sensor pairing (i.e., varying patterns between conditions). This aspect is not always examined directly in traditional image fusion studies because the emphasis is typically on the fused image. Nonetheless, single-band sensor choices are very important. Given that different spectral bands highlight different components of the image, it is not surprising that changes to single-band combinations have an impact on the effect of fused imagery on vision. With the differences in efficiency patterns over conditions in our studies, it is evident that this property has the potential to majorly influence image fusion success or failure and thus must always be considered when generalizing image fusion results.

Our experimental investigation as a whole established the foundational impacts of single-band and fused imagery on human efficiency, examining the general impact of fusion and its corresponding properties on the human visual system. Although our findings address the core goals of image fusion and enhancement, it should be noted that our patterns in results are specific to the simple experimental structure.

The Landolt C images utilized in our research, although redundant in overall shape, demonstrate the inherent elements present in single-band imagery and algorithmic fusion (e.g., “glow,” camera noise, edge “sharpness”/“blur”/“detail”) within a tightly controlled image capture environment. This basic stimulus was deliberately chosen to examine the impacts of single-band and algorithmic fusion combination at their most fundamental levels. Additionally, our task, intentionally chosen, encompassed a simple 1-of-8 choice of orientation. This provided strict focus and analysis to the effects of the image enhancements themselves.

The results from our experiment stand on their own for our simple stimuli and task and are important in showing that image fusion goals cannot always be assumed to be met over all implementations. However, the question remains as to whether these experimental findings will hold with more complex imagery, task, image capture conditions, and experimental parameters and which properties overall are most enhancing to the human visual system. It is entirely possible that introduction of further complexity to our experimental structure (e.g., stimuli of natural scenes, detection/search tasks) could result in increases in efficiency for image fusion.

Ideal observer analysis is uniquely designed to handle these types of questions, allowing for direct comparison of the impacts of each element and/or combinations of elements of the fusion problem space while accounting for changes in inherent information content due to stimulus, task, and other experimental design. We have shown ways in which human efficiencies can be compared over changes to imagery in sensor combination, fusion algorithm, and comparison of fused and unfused images. Ideal observer analysis can be augmented similarly to incorporate research involving more complex stimulus content. Additionally, the ideal observer can be adapted for a number of other task structures (e.g., detection, classification, discrimination) as well, through derivation of the decision rule for the given task constraints. However augmented, the use of this technique in relation to the study of image fusion must be implemented systematically to determine the driving forces for enhancement of human perception. The framework established here provides the structure for examining these questions. Additionally, the ideal observer itself can provide guidance for navigating the vast requirement of human data collection over various image enhancements in an experimentally obtainable manner.

### Framework and future directions

**Fig. 7:**
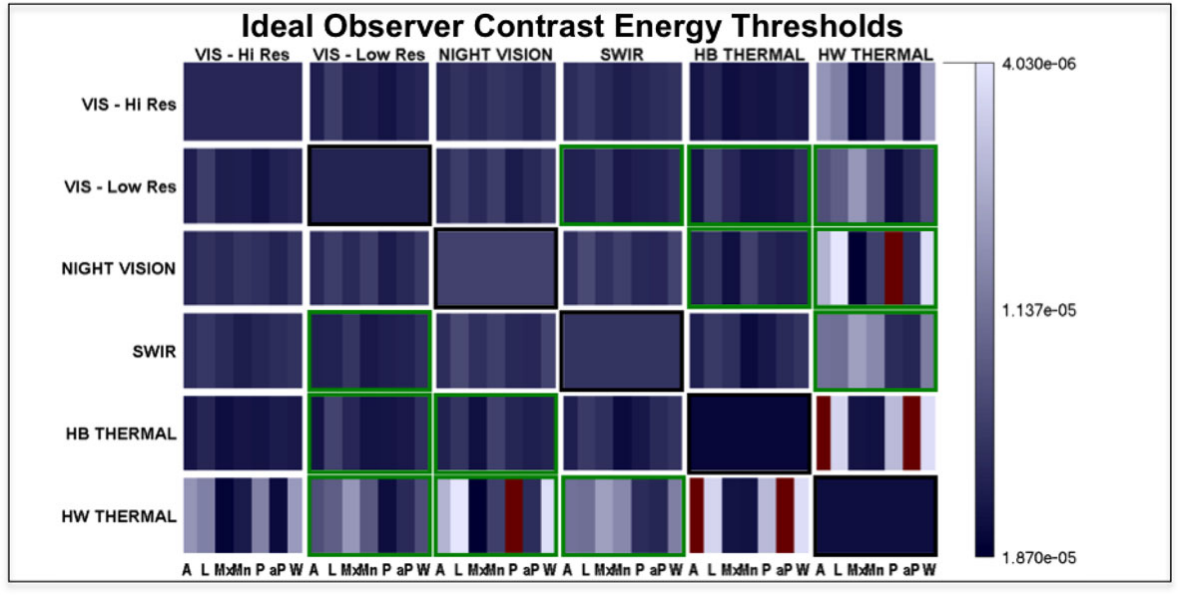
Ideal observer contrast energy thresholds over sets of single-band and fused imagery. Blocks on the main diagonal represent ideal performance using single-band imagery captured as labeled (i.e., *VIS-Hi Res*: visible camera with high resolution, *VIS-Low Res*: visible camera with low resolution, *Night Vision*, *SWIR*: short-wave infrared, *HB Thermal*: long-wave infrared image digitally inverted for hot-black imagery, *HW Thermal*: long-wave infrared hot-white imagery). All other off-diagonal blocks demonstrate ideal performance using fused imagery from the two designated components over the algorithms labeled on the bottommost axis (average *(A)*, Laplacian *(L)*, maximum *(Mx)*, minimum *(Mn)*, PCA *(P)*, adjusted PCA *(aP)*, and wavelet *(W)*). Off-diagonal blocks highlighted in green and diagonal blocks highlighted in black represent the conditions tested in the current paper’s experimental setup. Red blocks indicate conditions where reliable ideal observer thresholds could not be obtained

Recall that ideal observer performance is representative of the relative amount of information for the task across experimental properties and conditions. Given this, we can examine the variation in information over different fusion factors. For example, consider the heatmap in Fig. 7. This figure depicts the distribution of information (i.e., ideal observer performance) over sets of single-band and fused imagery in the 1-of-8 Landolt C orientation task used in the current paper. Within these results, we can examine particular patterns of information over the various combinations. For example, here we see that the on-diagonal conditions are in roughly the same range as many of the off-diagonal fused combinations (i.e., similar color in the heatmap). This demonstrates that our fusion conditions as a whole are not carrying *vastly greater* amounts of information than their single-band counterparts. Furthermore, we can note that individual fusion algorithms do not appear to produce consistent threshold values over conditions (i.e., we do not see clear vertically striped color patterns in the heatmap). This means that information variation is not affected by algorithm alone. Single-band images also differ from each in interesting ways (i.e., the values in the diagonal are not all the same color, but are close in some conditions). Exploring the impacts of these types of patterns can be of significant importance in understanding how each property or combination of properties affects information availability.

So how does this information heatmap help us tackle the problem space of image fusion in relation to *human* testing? As we have seen even within our own basic experiment, ideal observer performance does not always predict human results (e.g., the maximum algorithm in condition c0 is shown to carry the most information of the blocks, but humans use this information least efficiently). However, the similarities and differences in the distribution of information may provide valuable indicators as to how and what to experimentally test to parse patterns of influence on the human system. For example, consider the SWIR and visible stimuli in our experiment. We observed in our study that these stimulus types, when fused with hot-white thermal imagery (condition c0 and condition c3), produced similar patterns in efficiency data. Additionally, when fused together (condition c2), little to no significant differences were found between blocks in efficiency. Utilizing Fig. 7, we can see that the distributions in information over SWIR and visible (low resolution) combinations are roughly similar in general, whereas other sensors, like the hot-white thermal imagery, appear to produce much greater variation across conditions. Given that it would be practically infeasible to test all of the heatmap combinations on humans, these kinds of patterns are indicators of the best routes on which to systematically experiment across conditions. For example, here if we want to examine specific sensor influence, it may be a good choice to test a large number of hot-white thermal combinations given the potential for variations, whereas a conservative number of SWIR and visible combinations may suffice to hypothesize on their general influence.

**Fig. 8:**
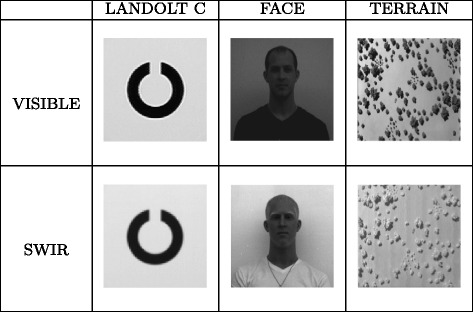
Imagery captured in SWIR and visible spectral bands. Landolt C images were used in the current experiment; face and landscape terrain board images were captured using the same cameras. Landscape terrain board images as in Neriani et al. (2008)

The image fusion problem space as a whole provides a large number of properties for future consideration in correspondence with human efficiency. Consider one of these properties in relation to our current stimuli. In Fig. 8 are examples of the SWIR and visible stimuli used in the current paper. These images visibly appear to be very similar to each other; however, with change to the stimulus content, substantial differences can be seen between the two sensors. Thus, an important future question is whether our results are indicative of general sensor impact or are possibly confined to our sensor-content-task link.

The examples surrounding Figs. 7 and 8 address specific changes to stimulus capture, combination, and content. However, factors such as task, image registration process and quality, collection conditions, and the like carry great potential to impact human perception as well. Additionally, although not demonstrated in our current image sets, there do exist conditions where image fusion itself has been inherently shown to provide impact to the viewer. That is, cases do exist where a fusion technique is applied to create an interpretable image from two completely uninterpretable component images (see Pavel, Larimer, & Ahumada 1991). Understanding these complex situations in relation to our base examination would also be of interest to the overall impact of fusion. These kinds of questions, as well as other variations to fusion factors, can all be explored using ideal observer analysis through adjustments and/or advancement of the framework established here.

## Conclusions

Given the nature of the ideal observer, the capability exists to examine the impact of information variation in ways that are not limited to our strictly defined basic experimental structure. As shown in our heatmap demonstration, information distributions can be derived across a number of fusion factors to inform the best options for human testing. We can then begin to systematically evaluate the impacts on human efficiency as demonstrated in our experimental applications.

The use of flexible tools that relate directly to human perception is essential when considering the general effects of image fusion and other image enhancements. These explorations are not only important in applied settings such as military and law enforcement research, but are also of great benefit in determining the cognitive impact of visual enhancements on human perception. With careful and strategic examination, future research will continue to aid our understanding of the overarching effects of this multidimensional problem space of image enhancement on human vision.

## Appendix: Fusion algorithms

The following provides a brief summary of the fusion algorithms utilized in this research. The Image Fusion Toolbox website (www.metapix.de/toolbox) provides further information regarding the detailed implementation of each technique. The interested reader is encouraged to explore the image fusion literature to compare and contrast these algorithms with alternative approaches, as these are representative of a subset of traditional algorithms used in image fusion.

### Minimum, maximum, and average fusion techniques

Minimum, maximum, and average fusion techniques rank among the simplest image fusion algorithms. In these algorithms, each image is represented as a matrix of pixel values. The function to fuse imagery between matrices can be described as a basic pixel-wise combination of corresponding positions in the individual component images. For example, in maximum fusion, the algorithm starts at the first pixel position (i.e., matrix cell) in both of the individual component images, determines the greatest pixel value between the two, and assigns this value to the first pixel position in the fused image. This process is repeated over all pixel positions until the full fused image is created. In minimum and average fusion, each corresponding pixel position is likewise evaluated with smallest and average values, respectively, assigned to the final fused image.

### Principal component analysis and adjusted PCA

Principal component analysis (PCA) is a general mathematical technique that transforms a set of potentially correlated variables into a set of linearly uncorrelated variables. It can be used for dimension reduction as well, by choosing a subset of the uncorrelated variables. This is done by performing a singular value decomposition (SVD) on the matrix of data, with each column representing a variable and each row representing a multivariate sample. SVD produces an orthogonal basis space, which is interpreted as a set of orthogonal variables called principal components. The first principal component is associated with the axis that captures the maximum variance. The second principal component is then constrained to be orthogonal to the first principal component while still capturing the most remaining variance. In image fusion, PCA is applied by treating each input image as a vector where the variables are the pixel values. In our application, because we are working with grayscale images, only the first principal component is utilized and assigned to the final fused image. Straight application of PCA to image fusion, as provided in the toolbox, can produce final image values that are outside of the viewable pixel range. Thus, an adjusted version of the PCA algorithm was utilized in our study to address this issue. Here principal component scores were transformed from a range of [–1,1], to the [0,1] domain to be properly displayed. More information on the PCA image fusion techniques can be found in Metwalli, Nasr, Allah, and El-Rabaie (2009) and similar fusion algorithm literature.

### Laplacian pyramid

Laplacian pyramid image fusion is a technique in which local operators of many scales but identical shape (as proposed by Burt and Adelson (1983)) are applied to the input images. Pixel-to-pixel correlations are first removed by subtracting a low-pass filtered copy of the image from the image itself. The result is a net data compression since the difference image has low variance and entropy, and the low-pass filtered image may be represented at reduced sample density. Further data compression is achieved by quantizing the difference image. These steps are then repeated to compress the low-pass image. Iteration of the process at appropriately expanded scales generates a pyramid data structure. The encoding process is equivalent to sampling the image with Laplacian operators of many scales, which tends to enhance salient image features.

### Discrete wavelet transform

The discrete wavelet transform (DWT) image fusion technique works by taking two spatially registered images with differing spatial resolutions and color content, combining the wavelet decomposition components from each input image, and then reconstructing the merged image by means of the inverse wavelet transform. The wavelet merger can employ a variety of wavelet bases. We utilized the Daubechies wavelet in our application. More information on the DWT can be found in Mallat (1996).
